# Clinical evidence for independent regulation of vitamin D by intestinal CYP24A1. Reply.

**DOI:** 10.1172/JCI191585

**Published:** 2025-04-15

**Authors:** Michaela A.A. Fuchs, Myles Wolf

**Affiliations:** 1 Division of Nephrology, Department of Medicine, Duke University School of Medicine, Durham, North Carolina, USA.; 2 Joan and Sanford I. Weill Department of Medicine, Weill Cornell Medicine, New York, New York, USA.

**Keywords:** Gastroenterology, Nephrology, Transcriptomics

**The authors reply:** Prior to the development of contemporary tools to modify gene expression at specific times and in specific organs, kidney cross-transplantation was used to isolate the effects of the kidneys versus other organs on the regulation of complex physiological systems (1, 2). In this technique, kidneys from mice with global deletion of a target gene would be transplanted into wild-type hosts, and wild-type kidneys would be transplanted into mice with global deletion of the target gene. Mechanistic inferences rested on determining whether the phenotype of interest followed the kidney or stayed with the host.

In their Letter to the Editor, Lemoine et al. report on a patient with longstanding, parathyroid hormone-independent hypercalcemia who received a kidney transplant from his healthy brother for treatment of end-stage kidney disease (ESKD) due to nephrocalcinosis of undiagnosed cause (3). Development of de novo posttransplant nephrolithiasis in the allograft suggested that the underlying disease was caused by systemic factors in the recipient rather than an intrinsic disease in the transplanted kidney. Subsequent genetic testing uncovered a homozygous inactivating mutation in *CYP24A1* in the patient, but no mutation in the kidney donor.

Acknowledging the inability to control for potential confounding factors, this “real world kidney cross-transplant experiment” in which a *CYP24A1^+/+^* kidney was transplanted into a *CYP24A1^–/–^* host broadly supports our finding that extrarenal *Cyp24a1* exerts systemic effects independent of renal *Cyp24a1* (4). The case report also raises important new questions. For example, the patient’s relatively elevated levels of 1,25-dihydroxy vitamin D during the posttransplant period contrast with our findings in mice with intestine-specific deletion of *Cyp24a1*, in which vitamin D levels remained normal. Since the patient lacked functional CYP24A1 in all his native cells other than his kidney allograft, this finding raises the possibility that other nonrenal, nonintestinal sources of CYP24A1 might contribute to circulating 1,25-dihydroxyvitamin D. Based on the welcome and important contribution by Lemoine et al., further studies are needed to understand other potential tissue-specific effects of *Cyp24a1*, including in the bone, parathyroid glands, and perhaps other organs.

## Version 1 04/15/2025

Electronic publication
